# Immunotherapies in Genitourinary Oncology: Where Are We Now? Where Are We Going?

**DOI:** 10.3390/cancers13205065

**Published:** 2021-10-10

**Authors:** Albert Jang, David M. Adler, Grant P. Rauterkus, Mehmet A. Bilen, Pedro C. Barata

**Affiliations:** 1 Deming Department of Medicine, Tulane University School of Medicine, New Orleans, LA 70112, USA; ajang@tulane.edu; 2 Section of Hematology and Medical Oncology, Deming Department of Medicine, Tulane University School of Medicine, New Orleans, LA 70112, USA; dadler1@tulane.edu; 3 Tulane University School of Medicine, New Orleans, LA 70112, USA; grauterk@tulane.edu; 4 Department of Hematology and Oncology, Winship Cancer Institute of Emory University School of Medicine, Atlanta, GA 30322, USA; mehmet.a.bilen@emory.edu; 5 Tulane Cancer Center, New Orleans, LA 70112, USA

**Keywords:** immunotherapy, immune checkpoint inhibitor, bladder cancer, urothelial cancer, kidney cancer, renal cell carcinoma, prostate cancer, testicular cancer, penile cancer, adrenocortical carcinoma

## Abstract

**Simple Summary:**

Genitourinary malignancies include cancers along the urinary tract and the male reproductive tract, encompassing the adrenal glands, kidneys, bladder, prostate, and testicles. Immunotherapy, which treats cancer by using the immune system to attack malignant cells, has historically been successful in treating some types of genitourinary cancers, especially of the bladder and kidney. In the past decade, a more precise method of immunotherapy, known as immune checkpoint inhibition, has gained popularity as it enhances the immune system’s ability to recognize and destroy tumor cells. Several immune checkpoint inhibitors have achieved success in patients with advanced genitourinary cancers. This review provides a brief overview of traditional immunotherapies, focuses on how immune checkpoint inhibitors have achieved success in patients with advanced cancers, and investigates the role for immunotherapy in genitourinary malignancies in the future.

**Abstract:**

For decades, limited options existed to treat metastatic genitourinary cancers, including treatment options that could be classified as immunotherapy. Historically, immunotherapy centered on systemic cytokines for the treatment of metastatic kidney cancer, which had several adverse effects, as well as the Bacillus Calmette–Guérin vaccine for non-metastatic bladder cancer. Within the past decade, advances in immunotherapy have led to several approvals from the United States Food and Drug Administration, particularly in the field of immune checkpoint inhibition. Immune checkpoint inhibitors (ICIs) are now being used extensively to treat multiple solid tumors, including kidney and bladder cancers, and they are also being tested in many other cancers. Despite encouraging data from phase 2/3 clinical trials, less is known about biomarkers that may predict better response to ICIs. The effect of ICIs in genitourinary cancers is heterogeneous, with some tumor types having little clinical data available, or ICIs having limited activity in other tumors. In this review, we briefly discuss approved immunotherapy agents prior to the time of ICIs. Then, given the emergence of this class of agents, we summarize the several important ICIs and the clinical trials that led to their approval. Finally, we mention ongoing and future clinical trials.

## 1. Introduction

Genitourinary (GU) malignancies affect many people worldwide, with millions of new cases diagnosed annually. According to the World Health Organization’s Global Cancer Observatory, in the year 2020, there were over 1,400,000 new diagnoses of prostate cancer, over 550,000 new cases of bladder cancer (in this article, interchangeable with urothelial carcinoma, which accounts for 90% of bladder cancer cases), and over 400,000 new cases of kidney cancer (in this article, interchangeable with renal cell carcinoma, which accounts for 90% of kidney cancer cases) [1]. Testicular cancer, a less common cancer, was diagnosed in over 70,000 people that same year [1]. Adrenocortical carcinoma is an even rarer tumor, with an estimated incidence of 1.02 per one million people [2].

In recent decades, our understanding of cancer and its pathophysiology has evolved. It has been well established that cancer cells have developed multiple mechanisms to avoid the immune system. This includes downregulation of antigen-presenting complexes via the Major Histocompatibility Complex (MHC) class 1 pathway in tumor cells [3], as well as suppressed host immune activation via regulatory T cells [4]. Multiple cytokines produced by tumors in large quantities, such as transforming growth factor beta (TGF-β), can also dampen the host immune response by preventing organic T-cell and NK-cell response [5]. By interfering with pathways in the tumor microenvironment, immune checkpoint inhibitors (ICIs) allow the immune system to “ramp up” and effectively target malignant cells. This article delves into the role of immunotherapy in the treatment of GU malignancies: specifically urothelial, renal, prostate, and other rarer tumors. We will review established molecular targets for GU immunotherapies as well as explore up-and-coming targets for disease treatment. We summarize selected clinical trials that are complete, those that are still in progress, and those that are about to begin.

## 2. Immune Checkpoint Inhibitors

The concept of immune checkpoint inhibition began with the discovery of genes encoding cytotoxic T-lymphocyte-associated protein 4 (CTLA-4) [6] and programmed cell death protein 1 (PD-1) [7] at the end of the 20th century. Both products are expressed on T cells; CTLA-4 binds to B7 ligand and PD-1 binds to programmed death ligand-1 (PD-L1). The downstream effect of these interactions suppresses the immune system through many mechanisms including diminishing T-cell immune response, decreasing the production of inflammatory cytokines, and increasing activity of regulatory T cells [8]. Cancer cells can take advantage of this signaling, allowing tumors to develop while avoiding attack from the immune system. The goal of immune checkpoint inhibition is to prevent the suppression of the immune system, resulting in its stimulation to destroy tumor cells.

A handful of ICIs have been approved for use in various malignancies by the Food and Drug Administration since 2011; these include one CTLA-4 inhibitor (ipilimumab), three PD-1 inhibitors (nivolumab, pembrolizumab, and cemiplimab), and three PD-L1 inhibitors (atezolizumab, avelumab, and durvalumab) [9]. Six of these agents have been successfully applied to various GU cancers as well, with all having received either full or accelerated approval in recent years (Table 1). Several ICIs targeting other pathways are currently in the developmental stages [10].

## 3. Bladder/Urothelial Cancer

The use of immunotherapy in bladder cancer has been well studied. It began with approval of the Bacillus Calmette–Guérin (BCG) vaccine in 1990, which produced a robust immune response against bladder tumors. However, this malignancy remains difficult to treat, especially after it has metastasized. The advancement of ICIs has revolutionized treatment for bladder cancer. Several ICIs have been successfully used in different stages of bladder cancer, resulting in approval by the FDA for various stages and conditions (Table 2).

### 3.1. Non-Muscle-Invasive Bladder Cancer (NMIBC)

#### 3.1.1. BCG

BCG was developed early in the 20th century as a live-attenuated vaccine that used *Mycobacterium bovis* against *Mycobacterium tuberculosis* to prevent clinical tuberculosis. BCG successfully inhibited tumor growth in the 1950s in mice that were infected with the vaccine [28]. Morales et al. [29] successfully used vesical and intradermal BCG in humans with superficial bladder cancer in the 1970s, which led to a successful prospective clinical trial [30]. The FDA approved intravesical BCG for patients with superficial bladder tumors in 1990, making it one of the most successful immunotherapy agents of the 20th century. It is thought that BCG causes innate and adaptive immune activation through cytokine and chemokine secretion, which leads to the recruitment of various immune cells to the bladder tumor site, resulting in death of bladder cancer cells [31]. BCG remains standard first-line treatment for high-risk NMIBC.

#### 3.1.2. ICIs for NMIBC

Despite the success of BCG, up to 40% of patients with NMIBC fail this treatment [32]. These patients have limited options, including radical cystectomy, chemotherapy, or clinical trials with novel methods such as chemoablation and oncolytic viruses [32]. ICIs demonstrated promise in these patients as well. 

The phase 2 trial KEYNOTE-057 (NCT02625961) enrolled patients with NIMBC who failed BCG to receive pembrolizumab [11]. After a median follow-up of 36.4 months, analysis of 96 patients showed a complete response rate (CRR) in 39 patients (40.6%) and median duration of response of 16.2 months (95% CI, 6.7–36.2), with no patients having progressed to muscle-invasive or metastatic disease. Based on this trial, pembrolizumab was approved in January 2020 for high-risk NMIBC with carcinoma in situ, with or without papillary tumors unresponsive to BCG therapy. KEYNOTE-676 (NCT03711032) is a phase 3 trial designed to expand on the findings from KEYNOTE-057 [33]. It is currently recruiting, and the primary outcome measures CRR and event-free survival.

Atezolizumab is another ICI that was used in BCG-unresponsive NMIBC in the phase 2 trial SWOG S1605 (NCT02844816). Preliminary results were reported at the 2020 ASCO Meeting. Among 73 patients with carcinoma in situ (CIS), there was a complete response detected in 30 patients (41.1%) at 3 months and 19 (26.0%) patients at 6 months [34]. Further follow-up data were provided at the 2021 ASCO Meeting, in which 74 patients with CIS had an 18 month event-free survival rate of 17% (90% CI, 9–25), while for all 128 patients (including non-CIS), the 18 month event-free survival rate was 29% (90% CI, 22–36) [35]. Despite the data suggesting similar activity to pembrolizumab in this setting, it did not meet its primary endpoints and thus has not been approved by the FDA.

### 3.2. Muscle-Invasive Bladder Cancer (MIBC)

#### 3.2.1. Neoadjuvant Therapy for MIBC

MIBC is treated with radical cystectomy [36]. Neoadjuvant cisplatin-based chemotherapy regimens have traditionally improved survival outcomes [37]. Immunotherapy is being explored as an alternative for cisplatin-ineligible patients in the neoadjuvant setting, though no agents are currently FDA approved for this indication. Initial findings from the phase 2 PURE-01 trial (NCT02736266) showed that 21 of 50 patients (42%) who received 3 courses of pembrolizumab before radical cystectomy resulted in pT0 [38]. On extended follow-up, event-free survival rate at 24 months was 71.7% (95% CI, 62.7–82.0) and recurrence-free survival rate was 39.3% (95% CI, 19.2–80.5), suggesting that pembrolizumab may make a favorable neoadjuvant choice compared to standard chemotherapy [39]. In the phase 2 trial ABACUS (NCT02662309), two cycles of atezolizumab were given before cystectomy, and among 88 patients with evaluable data, 27 had a pathologic complete response (31%; 95% CI, 21–41), which met initial primary endpoint goals [40]. These results support further investigation and validation of the role of ICIs in this setting.

#### 3.2.2. Adjuvant Therapy for MIBC

Unlike standard neoadjuvant therapy for MIBC, less is known regarding standard adjuvant chemotherapy due to poor clinical outcomes [41]. Phase 3 trials evaluating the use of adjuvant therapy with different ICIs are currently ongoing for MIBC patients. 

In the IMvigor010 (NCT02450331) trial, 809 patients with MIBC who underwent radical cystectomy or nephroureterectomy were randomized to either adjuvant atezolizumab or observation [42]. There was no significant difference in median disease-free survival in the atezolizumab versus observation group (19.4 months vs. 16.6 months; HR 0.89; 95% CI, 0.74–1.08; *p* = 0.24), while serious adverse effects occurred for nearly double the patients who received atezolizumab compared to the observation group (31% vs. 18%). As these results were somewhat disappointing, they discourage the use of atezolizumab in this setting.

In contrast, the CheckMate 274 trial (NCT02632409) evaluated adjuvant nivolumab versus placebo for 709 patients with muscle-invasive urothelial carcinoma who underwent radical cystectomy [12]. The median disease-free survival was 20.8 months (95% CI, 16.5–27.6) for patients who received nivolumab versus 10.8 months (95% CI, 8.3–13.9) for patients who received placebo, with 6 month survival/disease-free rate of 74.9% versus 60.3%, respectively (HR 0.70; 98.22% CI, 0.55–0.90; *p* < 0.001). The non-urothelial tract recurrence-free survival was 22.9 months (95% CI, 19.2–33.4) versus 13.7 months (95% CI, 8.4–20.3), with 6 month survival/recurrence-free rate of 77.0% versus 62.7%, respectively (HR 0.72; 95% CI, 0.59–0.89). For PD-L1-positive patients (defined as tumor PD-L1 expression of ≥1%), there was also a statistically significant advantage in survival at 6 months (75.3% vs. 56.7%; HR 0.55; 95% CI, 0.39–0.79). These results with nivolumab were very promising, and the FDA approved nivolumab in this setting in August 2021.

Other important studies are ongoing, such as the phase 3 trial AMBASSADOR (NCT03244384) which is evaluating pembrolizumab versus observation.

#### 3.2.3. Bladder-Sparing Treatments Involving ICIs in MIBC

The 2021 ASCO Meeting provided new phase 2 clinical trials involving ICIs focusing on bladder-sparing techniques. In HCRN GU16-257 (NCT03558087), 76 patients with MIBC underwent treatment with gemcitabine, cisplatin, and nivolumab followed by clinical restaging; 31 of 64 patients had complete remission from the regimen, and 30 patients who opted not to undergo cystectomy received additional nivolumab and were alive at the time of data cutoff [43]. In another phase 2 trial (NCT02621151), 54 patients received one dose of pembrolizumab, followed by maximal transurethral resection of the bladder tumor (TURBT), followed by pembrolizumab again along with whole bladder radiation and gemcitabine. Forty-two patients (85%) completed the study and the estimated one-year bladder-intact disease-free survival rate was 77% [44]. In IMMUNOPRESERVE-SOGUG (NCT03702179), 32 patients received transurethral resection followed by durvalumab and tremelimumab, then normo-fractionated external beam radiation therapy. Twenty-six patients (81%) achieved complete remission and 12 month disease-free survival rate was 73% (95% CI, 59–91) [45]. These studies show promising preliminary data for patients with worsening MIBC and for those who are not candidates for radical cystectomy.

### 3.3. Metastatic Bladder Cancer

#### 3.3.1. Frontline Maintenance Therapy for Locally Advanced or Metastatic Bladder Cancer in Patients Who Previously Received Cisplatin Chemotherapy

After patients receive first-line chemotherapy, some patients will receive maintenance therapy to prevent disease progression or recurrence. This strategy has been applied to several malignancies [46]. Changing the initial induction chemotherapy agent to another agent with a different mechanism of action, commonly known as “switch maintenance,” may avoid extra toxicities while providing synergistic disease benefit. The rationale for immune checkpoint inhibition as maintenance therapy after chemotherapy has been applied to different tumors including bladder cancer.

In the phase 3 trial JAVELIN Bladder 100 (NCT02603432), 700 patients with locally advanced or metastatic urothelial carcinoma (mUC) who did not progress after first-line platinum-based chemotherapy (gemcitabine plus cisplatin or carboplatin), were randomized to receive avelumab (*n* = 350) versus best supportive care (*n* = 350) [13]. Results were statistically and clinically meaningful for the patients who received avelumab in terms of median PFS (mPFS) (3.7 months vs. 2.0 months; HR 0.62; 95% CI 0.52–0.75) and median OS (mOS) (21.4 months vs. 14.3 months; HR 0.69; 95% CI, 0.56–0.86, *p* = 0.001). There was also significant benefit for the PD-L1+ group (based on study criteria) who received avelumab over best supportive care based on mPFS (5.7 months vs. 2.1 months; HR 0.56; 95% CI, 0.43–0.73) and one-year survival (79.1% vs. 60.4%; HR 0.56; 95% CI, 0.40–0.79; *p* < 0.001). Based on these results, avelumab was approved in June 2020 as a maintenance treatment in this setting. Of note, avelumab remains the first and only ICI approved in the front-line setting for cisplatin-eligible patients as maintenance therapy.

In a smaller phase 2 study, pembrolizumab achieved less success when used as switch maintenance after platinum-based chemotherapy in metastatic bladder cancer patients (NCT02500121) [47]. Of 108 patients enrolled in the trial, 55 received pembrolizumab and 53 received placebo. ORR was 23% with pembrolizumab versus 10% with placebo. The mPFS was significantly longer for pembrolizumab maintenance compared to placebo (5.4 months vs. 3.0 months; HR 0.65, *p* = 0.04), but the mOS was not significantly different (22 months vs. 18.7 months; HR 0.91; 95% CI, 0.52–1.59). In this trial, there was no significant benefit for PD-L1-positive patients (based on combined positive score ≥ 10) for either treatment (*p* = 0.8 for PFS and *p* = 0.9 for OS).

A single-arm phase 2 trial (NCT01524991) enrolled 36 patients with mUC and no prior chemotherapy to two cycles of gemcitabine and cisplatin followed by four cycles of gemcitabine, cisplatin, and ipilimumab [48]. This small study was not successful in meeting its primary endpoint of the lower bound of 90% CI for one-year OS >60%, with one-year OS of 0.61 (lower bound 90% CI of 0.51). The mOS was 13.9 months (95% CI, 10.5–23.4), and mPFS was 7.9 months (95% CI, 6.4–9.9). 

#### 3.3.2. Frontline Use in Combination with Chemotherapy

The combination of ICIs with chemotherapy, which has been successfully implemented in other solid tumors such as non-small-cell lung cancer [49], has been tested in mUC.

The phase 3 trial IMvigor130 (NCT02807636) included 1213 patients that were divided into three arms: atezolizumab with chemotherapy (*n* = 451), atezolizumab monotherapy (*n* = 362), and placebo with chemotherapy (*n* = 400) [50]. Comparing the cohorts of atezolizumab with chemotherapy and placebo with chemotherapy, mPFS was 8.2 months versus 6.3 months (HR = 0.82; 95% CI, 0.70–0.96; *p* = 0.007), which met one prespecified efficacy endpoint. However, mOS was 16.0 months versus 13.4 months (HR 0.83; 95% CI, 0.69–1.00; *p* = 0.027) which did not meet the other prespecified efficacy endpoint. The mOS for atezolizumab monotherapy also did not statistically differ from the placebo/chemotherapy cohort (HR 1.02; 95% CI, 0.83–1.24).

The phase 3 trial KEYNOTE-361 (NCT02853305) included 1010 patients with mUC who did not receive prior systemic therapy. They were randomized to receive pembrolizumab with chemotherapy (*n* = 351), pembrolizumab only (*n* = 307), or chemotherapy only (*n* = 352) [51]. Between the pembrolizumab with chemotherapy versus chemotherapy only group, the mPFS (8.3 months vs. 7.1 months; HR 0.78; 95% CI, 0.65–0.93; *p* = 0.0033) and mOS (17.0 months vs. 14.3 months; HR 0.86; 95% CI, 0.72–1.02, *p* = 0.0407) did not reach statistical significance based on pre-specified *p*-value cutoffs.

The phase 2 trial INDUCOMAIN (NCT03390595) evaluated 85 cisplatin-ineligible patients with untreated metastatic urothelial carcinoma; 42 patients received two cycles of induction therapy of avelumab followed by six cycles of avelumab with carboplatin-gemcitabine followed by two weeks of maintenance therapy with avelumab, compared to 43 patients who received six cycles of carboplatin-gemcitabine [52]. Preliminary results presented at the European Society of Medical Oncology (ESMO) Virtual Congress 2020 showed that the avelumab cohort performed worse than the standard chemotherapy cohort based on ORR (57% vs. 53%, *p* = 0.73), mPFS (6.9 months vs. 7.4 months, *p* = 0.712), and mOS (10.5 months vs. 13.2 months, *p* = 0.264).

Results to date have been disappointing, but more phase 3 trials of frontline ICI and chemotherapy combination regimens for mUC are underway. NILE (NCT03682068) is recruiting 1434 patients to three arms: a combination of tremelimumab, durvalumab, and standard of care (SoC) chemotherapy, durvalumab and SoC chemotherapy, or SoC chemotherapy alone. CheckMate 901 (NCT03036098) is recruiting 1290 patients that will also be assigned to different treatment arms: nivolumab and ipilimumab, nivolumab and SoC chemotherapy, or SoC chemotherapy alone.

Overall, ICIs combined with chemotherapy have not been successful in the setting of mUC. Pembrolizumab, nivolumab, and atezolizumab individually combined with chemotherapy have not produced statistically significant benefits in mOS and mPFS compared to chemotherapy alone when used as first-line therapy.

#### 3.3.3. Frontline Use with Combination of Two ICIs

Combination immunotherapy in the frontline setting is another option for mUC and has been evaluated in multiple trials. The phase 3 trial DANUBE (NCT02516241) included 1032 previously untreated, locally advanced and mUC patients that were divided into three arms: durvalumab plus tremelimumab (*n* = 342), durvalumab monotherapy (*n* = 346), and chemotherapy (*n* = 344), and patients were followed for a median 41.2 months [53]. Neither of the primary endpoints (mOS in both the high PD-L1 cohort and overall intention-to-treat group) was met in the study, as chemotherapy appeared to be more favorable than immunotherapy. In the population with high PD-L1 expression (as defined by the authors of the study), mOS was not statistically different for the durvalumab group versus chemotherapy group (14.4 months vs. 12.1 months; HR 0.89; 95% CI, 0.71–1.11; *p* = 0.30). For the overall intention-to-treat group, mOS was not statistically significant between the durvalumab and tremelimumab combination versus the chemotherapy group (15.1 months vs. 12.1 months; HR 0.85; 95% CI, 0.72–1.02; *p* = 0.08). In the intention-to-treat population, mPFS was the longest for the chemotherapy group at 6.7 months (95% CI, 5.7–7.3) compared to the durvalumab group (2.3 months; 95% CI, 1.9–3.5) and the durvalumab plus tremelimumab group (3.7 months; 95% CI, 3.4–4.8). 

Overall, the combination of durvalumab and tremelimumab provided disappointing results. Another immunotherapy combination, nivolumab and ipilimumab, has been successfully used in the frontline setting for advanced RCC [54], advanced melanoma [55], and advanced non-small-cell lung cancer [56]. This regimen is currently being evaluated in mUC in the phase 3 trial CheckMate 901 (NCT03036098), as mentioned above in the prior section.

#### 3.3.4. Frontline Use for Locally Advanced or Metastatic Cisplatin-Ineligible Patients

Different ICIs have been tested in the front line setting for cisplatin-ineligible patients in small, proof-of-concept studies. In cohort 1 of the phase 2 trial IMvigor210 (NCT02951767), 119 previously untreated cisplatin-ineligible patients with mUC received atezolizumab and had a mPFS of 2.7 months (95% CI, 2.1–4.2), mOS of 15.9 months (95% CI, 10.4–NE), and 23% ORR (95% CI, 16–31) at a median follow-up of 17.2 months [14]. Atezolizumab was granted accelerated approval in April 2017 based on these results. Updated follow-up time to a median of 29 months was presented at the 2018 ASCO Meeting, and results of mOS (16.3 months) and ORR (24%) were consistent with prior data [15].

In the phase 2 trial KEYNOTE-052 (NCT02335424), 89 of 370 patients (24%; 95% CI, 20–29) with no prior systemic chemotherapy received pembrolizumab and achieved an objective response [16]. Pembrolizumab was granted accelerated approval in May 2017 for use in these patients. With a minimum follow-up of two years, pembrolizumab continued to be effective in terms of ORR (28.6%; 95% CI, 24.1–33.5) and mOS (11.3 months; 95% CI, 9.7–13.1) [17]. The phase 3 trial KEYNOTE-361 (NCT02853305) included 1010 patients with advanced UC who received no prior systemic therapy were randomized to receive pembrolizumab with chemotherapy (*n* = 351), pembrolizumab only (*n* = 307), or chemotherapy only (*n* = 352). The preliminary data presented at the ESMO Virtual Congress 2020 showed that PFS and OS did not reach statistical significance between the pembrolizumab and chemotherapy group versus chemotherapy only [57]. In spite of KEYNOTE-361 and IMvigor310 (as mentioned in a prior section), at a recent Oncologic Drugs Advisory Committee meeting, that both pembrolizumab and atezolizumab retain accelerated approval as first-line therapy for advanced UC [58]. In August 2021, the FDA granted pembrolizumab full approval, given the lack of approved therapies in this frontline setting [59]. 

Additionally, pembrolizumab is also being tested in combination with enfortumab vedotin (EV), an antibody–drug conjugate that binds to nectin-4, as first-line therapy in cisplatin-ineligible patients in the phase 1/2 trial EV-103/KEYNOTE-869 (NCT03288545). Results presented at the 2020 ASCO Meeting showed 45 patients with a median of 9 cycles of EV with pembrolizumab had an ORR of 73.3% (95% CI, 58.1–85.4), 12 month OS rate of 81.6% (95% CI, 62.0–91.8), and a mPFS of 12.3 months (95% CI, 7.98–NR) [18]. These results led the FDA to grant this combination as breakthrough designation therapy in February 2020.

#### 3.3.5. Second-Line Therapy for Locally Advanced or Metastatic Bladder Cancer Patients after Cisplatin Chemotherapy

Before immune checkpoint inhibition, prognosis was grim for patients who relapsed after first-line platinum therapy due to a lack of available options [60], until ICIs provided renewed hope and improved patient outcomes in the second-line setting [61]. Several trials have been conducted with different ICIs as both monotherapy and as combination therapy. 

In cohort 2 of IMvigor210 (NCT02108652), 310 patients with locally advanced or metastatic UC who were previously treated with cisplatin-containing chemotherapy and progressed were then administered atezolizumab. The objective response rate (ORR) was 15% (95% CI, 11–19), mPFS was 2.1 months (95% CI, 2.1–2.1), and mOS was 7.9 months (95% CI, 6.6–9.3) [19]. This resulted in accelerated approval for atezolizumab in May 2016 for this indication. The confirmatory phase 3 study IMvigor211 randomized 931 patients with metastatic UC who progressed after platinum-based chemotherapy. Patients were then treated with atezolizumab (*n* = 467) versus physician’s choice of chemotherapy (*n* = 464). There was no survival benefit for atezolizumab in the patients with ≥5% tumor-infiltrating immune cells positive for PD-L1 (primary endpoint) with a mOS of 11.1 months versus 10.6 months (HR 0.87; 95% CI, 0.63–1.21; *p* = 0.41) [62]. Ultimately, the approval of atezolizumab as a second-line treatment was withdrawn in 2021 [63].

The phase 2 trial CheckMate 275 (NCT02387996) evaluated nivolumab in patients with locally advanced or mUC with disease progression during, or following, platinum-containing chemotherapy, or with disease progression within 12 months of neoadjuvant or adjuvant treatment with a platinum-containing chemotherapy [20]. Among 265 patients who received nivolumab, 52 (19.6%; 95% CI, 15.0–24.9) had a confirmed objective response. This led to accelerated approval for nivolumab for this indication in February 2017. On extended follow-up of at least 33.7 months, nivolumab continued to maintain its effectiveness [21].

In the phase 3 trial KEYNOTE-045 (NCT02256436), 542 patients with locally advanced or mUC with disease progression during, or following, platinum-containing chemotherapy, or within 12 months of receiving neoadjuvant or adjuvant treatment with platinum-containing chemotherapy, were randomized to receive pembrolizumab (*n* = 270) or further chemotherapy (*n* = 272) [22]. The mOS (10.3 months vs. 7.4 months; HR 0.73; 95% CI, 0.59–0.91; *p* = 0.002) and ORR (21.1% vs. 11.4%, *p* = 0.001) both favored the group that received pembrolizumab. However, there was no significant difference in PFS between the two groups (HR 0.98; 95% CI, 0.81–1.19; *p* = 0.42). Based on the mOS and ORR, pembrolizumab received approval in this treatment setting in May 2017. With an extended median follow-up to 27.7 months, data continued to be consistent for OS and PFS, with maintained OS benefit and no statistical difference between PFS. Yet, at the 24 month cutoff, the PFS value was higher for the pembrolizumab group compared to the chemotherapy group (12.4% vs. 3.0%) [23]. Of note, pembrolizumab has been the only ICI to show a survival advantage compared with cytotoxic chemotherapy in this setting. 

In May 2017, avelumab received accelerated approval as second-line treatment in this group of patients, based on promising results from the dose-escalation phase 1 trial of the cohort of UC patients enrolled in JAVELIN Solid Tumor (NCT01772004) [24]. On extended follow-up of a minimum of 24 months, for 242 patients with mUC who had failed platinum therapy, the confirmed ORR was 16.5% (95% CI, 12.1–21.8), mPFS was 1.6 months (95% CI, 1.4–2.7), and mOS was 7.0 months (95% CI, 5.9–8.5) [25]. In that same month, the FDA also granted durvalumab accelerated approval as second-line treatment for this group of patients based on a phase 1/2 trial Study 1108 (NCT01693562) [26,27]. In extended follow-up of 191 UC patients (182 with prior platinum treatment), ORR was 17.8% (34 of 191; 95% CI, 12.7–24.0), mPFS was 1.5 months (95% CI, 1.4–1.9 months), and mOS was 18.2 months (95% CI, 8.1–NE) [27]. However, based on the DANUBE trial not meeting its primary endpoints as mentioned earlier, this accelerated approval designation for durvalumab was withdrawn in 2021 [64].

ICI combination has been investigated in the refractory disease setting. For example, the phase 1/2 trial CheckMate 032 (NCT01928394), which tested the safety and efficacy of nivolumab with and without ipilimumab, contained a cohort of 274 locally advanced or mUC patients that progressed on platinum chemotherapy were then treated with nivolumab monotherapy at a dose of 3 mg/kg (*n* = 78), nivolumab 3 mg/kg with ipilimumab 1 mg/kg (*n* = 104), or nivolumab 1 mg/kg with ipilimumab 3 mg/kg (*n* = 92) [65]. In the most recent data presented at the ESMO Virtual Congress 2020, the nivolumab 1 mg/kg with ipilimumab 3 mg/kg demonstrated superior clinical outcomes based on ORR, mPFS, and mOS, and patients with PD-L1 expression of ≥1% showing even better outcomes compared to patients with PD-L1 expression <1% [66].

In summary, within a one-year time span, atezolizumab, avelumab, nivolumab, pembrolizumab, and durvalumab were all approved by the FDA for second-line therapy, largely based on promising data from the phase 2 trials for patients in this stage. However, as the phase 3 data now available have been less encouraging than was initially expected for some of these agents, specifically for atezolizumab (IMvigor211) and durvalumab (DANUBE), there has been voluntary removal from drug manufacturers for this designation. Pembrolizumab remains the ICI of choice based on level 1 evidence.

### 3.4. Variant Histologic Subtypes of Urothelial Carcinoma and Response to ICIs

Most bladder cancer histology is pure urothelial carcinoma (PUC); however, there has been increased recognition of many other histological subtypes, collectively known as variant urothelial carcinomas (VUC) [67]. VUC may be more aggressive than PUC, and patients with VUC appear to have a worse prognosis [68,69]. Evidence for optimal treatments is lacking in VUC, as patients with VUC are generally excluded from clinical trials. In a retrospective multi-institutional study, 286 patients with PUC responded to ICIs at similar rates to 120 patients with VUC based on ORR (28% vs. 29%, *p* = 0.90), mOS (11.0 months vs. 10.1 months, *p* = 0.60), and mPFS (4.1 months vs. 5.2 months, *p* = 0.43), while patients with VUC with neuroendocrine features had the lowest PFS and OS [70]. More prospective clinical data are necessary to better evaluate ICIs in patients with VUC.

## 4. Kidney Cancer

The most common type of kidney cancer is renal cell carcinoma (RCC), which can be categorized as clear cell (cc) or non-clear cell (ncc) based on histology. There are now several ICI options available to treat RCC (Table 3). However, in the past, few therapies existed to treat metastatic RCC for many decades due to its lack of sensitivity to chemotherapy and radiation [71]. Systemic cytokine therapy was used to treat RCC prior to the development of targeted therapies and ICIs. Interleukin-2 (IL-2) and Interferon (IFN) alfa-2a were two cytokines that received FDA approval for metastatic RCC. 

High-dose IL-2 was approved as a treatment for metastatic RCC by the FDA in 1992. In one of the earliest published studies of high-dose IL-2, among 149 patients with metastatic RCC that had failed prior treatment, there was an overall response of 20% [72]. A randomized phase 3 trial compared high-dose IL-2 with subcutaneous IL-2 combined with IFN alfa-2b; overall response rate was 23.2% versus 9.9% (*p* = 0.018) and durable complete response was 7.4% versus 0% in favor of high-dose IL-2. There was no significant difference in mPFS (*p* = 0.082) or OS (*p* = 0.211), although the data also favored high-dose IL-2 [73]. Overall, high-dose IL-2 therapy was associated with severe adverse effects such as capillary leak syndrome which historically limited its use to young and fit patients. 

IFN alfa-2a received FDA approval in 2009 to treat metastatic RCC when combined with bevacizumab based on the results of the randomized phase 3 trial AVOREN [74,75]. This study enrolled 649 patients with previously untreated metastatic RCC to receive IFN alfa-2a with bevacizumab or IFN alfa-2a with placebo. Median duration of PFS for interferon with bevacizumab was significantly longer compared to the control group (10.2 months vs. 5.4 months; HR 0.63; 95% CI, 0.52–0.75; *p* = 0.0001), and ORR was 31% to 13% in favor of interferon with bevacizumab (*p* = 0.001) [74]. The mOS between the two groups showed no statistically significant benefit for interferon with bevacizumab [75]. 

### 4.1. Contemporary Frontline Therapies with ICIs

#### 4.1.1. Combination of Two ICIs

To date, the only combination of two ICIs as frontline therapy for RCC approved by the FDA (April 2018) is ipilimumab plus nivolumab, specifically for intermediate- and poor-risk previously untreated advanced RCC based on the results of the phase 3 trial CheckMate 214 (NCT02231749) [76]. This combination marked the first ICIs to be approved in the frontline setting for metastatic RCC. For intermediate- and poor-risk patients, 18 month overall survival rate was 75% (95% CI, 70–78) for the immunotherapy combination versus 60% (95% CI, 55–65) for sunitinib; ORR was 42% versus 27% (*p* < 0.001), and CRR was 9% versus 1%. In favorable-risk patients, the immunotherapy combination fared worse than sunitinib in terms of mPFS (15.3 months vs. 25.1 months; HR 2.18; 99.1% CI, 1.29–3.68; *p* < 0.001) and ORR (29% vs. 52%, *p* < 0.001). For an extended follow-up minimum of four years, the mPFS and mOS remained advantageous for ipilimumab plus nivolumab over sunitinib for the intermediate- and poor-risk populations, but less effective than sunitinib for the favorable-risk population [77].

#### 4.1.2. Combination of ICI with Tyrosine Kinase Inhibitor

Many ICIs have now been approved by the FDA as front-line therapy for metastatic ccRCC. This includes ICIs used in combination with a tyrosine kinase inhibitor (TKI) or an antibody that targets vascular endothelial growth factor (VEGF) or its associated receptor (VEGF-R). These therapies include pembrolizumab with axitinib, atezolizumab with bevacizumab, avelumab with axitinib, pembrolizumab with lenvatinib, and nivolumab with cabozantinib. Understanding angiogenesis has played a key role in the development of TKIs which target VEGF-R [87]. The combination of ICI and VEGF inhibitors together is based on the discovery that VEGF plays a role in immune suppression of the tumor microenvironment; hence, VEGF inhibition could provide a synergistic benefit with ICIs to reverse immune suppression and allow the immune system to more effectively destroy cancer cells [88,89,90]. Phase 3 clinical trials all compared these combinations with sunitinib monotherapy, which itself had been shown to be more effective than IFN as first-line therapy [91]. 

The phase 3 trial KEYNOTE-426 (NCT02853331) enrolled 861 advanced ccRCC patients to either pembrolizumab and axitinib (*n* = 432) or sunitinib (*n* = 429) [78]. The mPFS, 12 month overall survival, and ORR were all significantly better in the pembrolizumab-axitinib group compared to the sunitinib group, with benefit seen in all IMDC risk groups. Pembrolizumab with axitinib was approved in April 2019 based on the results. On extended follow-up (median 30.6 months), benefit for the pembrolizumab-axitinib cohort was maintained based on mPFS (15.4 months vs. 11.1 months; HR 0.71; 95% CI, 0.60–0.84; *p* < 0.0001) and mOS (NR vs. 35.7 months; HR 0.68; 95% CI, 0.55–0.85; *p* = 0.0003); however, for the International Metastatic RCC Database Consortium (IMDC) favorable subgroup, there was no statistically significant difference between pembrolizumab-axitinib versus sunitinib in terms of either mPFS (HR 0.79; 95% CI, 0.57–1.09; *p* = 0.078) or mOS (HR 1.06; 95% CI, 0.60–1.86; *p* = 0.58) [79]. 

The phase 3 trial CheckMate 9ER (NCT03141177) enrolled 651 patients, randomized to either nivolumab and cabozantinib (*n* = 323) or sunitinib (*n* = 328) [81]. At a median follow-up of 18.1 months, mPFS was 16.6 months versus 8.3 months in favor of cabozantinib and nivolumab over sunitinib (HR 0.51; 95% CI, 0.41–0.64; *p* < 0.001). OS at 12 months was 85.7% for cabozantinib and nivolumab compared to 75.6% for sunitinib (HR for death 0.60; 98.9% CI, 0.40–0.89; *p* = 0.001), and objective response was 55.7% versus 27.1% (*p* < 0.001). Nivolumab with cabozantinib was approved in January 2021 as first-line treatment based on this data.

The combination of lenvatinib and pembrolizumab together was provided breakthrough designation in January 2018 for RCC after results of the phase 1b/2 trial Study 111/KEYNOTE-146 (NCT02501096), in which an ORR was achieved in 21 of 30 RCC patients (70%; 95% CI, 50.6–85.3) [92]. Eight of the 30 patients (27%) had received at least two lines of treatment prior to the trial. Subsequently, the pivotal phase 3 trial CLEAR/KEYNOTE-581 (NCT02811861) compared pembrolizumab with lenvatinib (*n* = 355) to either lenvatinib with everolimus (*n* = 357) or sunitinib (*n* = 357) in patients who received no prior systemic therapy [80]. Lenvatinib with pembrolizumab compared to sunitinib showed both superior mPFS (23.9 vs. 9.2 months; HR 0.39; 95% CI, 0.32–0.49; *p* < 0.001) and OS (HR 0.66; 95% CI, 0.49–0.88; *p* = 0.005). Lenvatinib with everolimus had superior mPFS compared to sunitinib (14.7 vs. 9.2 months; HR 0.65; 95% CI, 0.53–0.80; *p* < 0.001), but there was no statistical difference for OS (HR 1.15; 95% CI, 0.88–1.50; *p* = 0.30). The FDA approved the combination of lenvatinib and pembrolizumab in August 2021.

The phase 3 trial JAVELIN Renal 101 (NCT02684006) enrolled 886 patients, randomized to either avelumab and axitinib (*n* = 442) or sunitinib (*n* = 444) [82]. In the trial, avelumab and axitinib were superior to sunitinib in terms of mPFS in the PD-L1+ subgroup and the overall intention to treat group. Avelumab plus axitinib received approval in May 2019 based on these results. Updated results with a minimum follow-up of 13 months continued to show patients had a significantly better mPFS when they received avelumab with axitinib (13.3 months vs. 8.0 months; HR 0.69; 95% CI, 0.57–0.83; *p* < 0.0001) [83]. This trial so far has not revealed significant differences in OS between groups (HR 0.796; 95% CI, 0.616–1.027; *p* = 0.0392), although OS data remain immature.

Finally, the phase 3 trial IMmotion151 (NCT02420821) enrolled 915 patients with ccRCC and sarcomatoid RCC, and it produced promising results [84]. Atezolizumab with bevacizumab (*n* = 454) demonstrated superior mPFS over sunitinib (*n* = 461) (HR 0.83; 95% CI, 0.70–0.97; *p* = 0.02), but there was no significant difference in mOS (HR 0.93; 95% CI, 0.76–1.14; *p* = 0.48). Long-term follow-up data for this trial also have not yet been reported. The FDA has not yet approved the combination of atezolizumab plus bevacizumab as a treatment regimen for advanced RCC.

So far, the combinations of pembrolizumab plus axitinib, pembrolizumab plus lenvatinib, and nivolumab plus cabozantinib (in addition to ipilimumab plus nivolumab) have demonstrated statistically significant improvement in mOS and mPFS over sunitinib, while avelumab plus axitinib and atezolizumab plus bevacizumab have demonstrated statistically significant improvement in mPFS only. With several options recently approved in the frontline setting, there is now debate over the optimal regimen to use. No trials exist that directly compare combinations of ICI with VEGF/VEGF-R inhibitors, and the differences in demographics among patients enrolled in these trials prevent direct comparisons retrospectively. Patients enrolled in CLEAR who received lenvatinib and pembrolizumab had similar or improved quality of life over sunitinib [93]. Notably, the patients enrolled in CheckMate 9ER [94] and CheckMate 214 [95] who received ICI combination had superior quality of life over sunitinib. All regimens showed superior activity in tumors with sarcomatoid and rhabdoid features. Ultimately, the optimal treatment regimen is based on multiple factors including risk group, tumor burden, location of metastases, efficacy endpoints and patient and physician preference. 

#### 4.1.3. ICI Monotherapy

Although combination immunotherapy and ICI synergy with an anti-angiogenic agent have demonstrated success, adverse effects that lead to dose adjustments, treatment interruption, and diminished quality of life are common concerns. Hence, the concept of ICI monotherapy is being trialed as an alternative for patients who likely will not tolerate combination therapy. Pembrolizumab was recently tested as monotherapy in the open-label phase 2 trial KEYNOTE-427 (NCT02853344), in clear cell (cohort A) [96] and non-clear cell (cohort B) RCC [97]. In cohort A (*n* = 110), the ORR was 36.4% (95% CI, 27.4–46.1), mPFS was 7.1 months (95% CI, 5.6–11.0), and 24 month overall survival rate was 70.8% (mOS not reached). From a safety perspective, pembrolizumab was favorably tolerated. Nivolumab has been tested as first-line monotherapy in the phase 2 studies of HCRN GU16-260 (NCT03117309) [98] and OMNIVORE (NCT03203473) [99], which produced modest results and had adverse effects similar to prior studies involving nivolumab. Retrospective experience with ICI monotherapy has reported similar outcomes [100]. While these are proof-of concept studies, they raise the question of whether ICI monotherapy is an effective treatment strategy. 

### 4.2. Second-Line Immunotherapy

Nivolumab was originally approved in November 2015 for patients with advanced RCC treated with prior anti-angiogenic therapy based on results of CheckMate 025 (NCT01668784), in which 406 patients received nivolumab and 397 received everolimus [85]. In the original study, mOS for nivolumab was 25.0 months (95% CI, 21.8–NE) versus 19.6 months for everolimus (95% CI, 17.6–23.1) with HR 0.73 (98.5% CI, 0.57–0.93; *p* = 0.002), while mPFS difference was not statistically significant different (4.6 months vs. 4.4 months; HR 0.88; 95% CI, 0.75–1.03; *p* = 0.11). In the updated median follow-up of 72 months, nivolumab continued to have superior OS (25.8 months vs. 19.7 months; HR 0.73; 95% CI, 0.62–0.85; *p* < 0.0001), and nivolumab had favorable PFS (HR 0.84; 95% CI, 0.72–0.99; *p* = 0.0331) [86].

The combination of lenvatinib and pembrolizumab as second-line therapy for 104 metastatic ccRCC patients, who previously progressed during, or following, ICI therapy was studied in the phase 2 expansion cohort of Study 111/KEYNOTE-146 (NCT02501096) [101]. For 91 patients with an evaluable response, the ORR was 51% (95% CI, 39.9–62.2). The mPFS for 103 patients was 11.7 months (95% CI, 9.5–NE). The study suggests that lenvatinib with pembrolizumab may warrant further investigation as a second-line option for relapsed metastatic ccRCC treated with prior PD-1/PD-L1 inhibitors. Other phase 3 trials investigating the addition of ICI to TKI in the refractory setting including TiNivo2 [102], which combines tivozanib and nivolumab, and CONTACT-03 (NCT04338269) [103], which combines atezolizumab and cabozantinib, are currently ongoing or being planned.

### 4.3. Adjuvant Therapy after Nephrectomy

Few options exist for patients with high-risk RCC who previously underwent curative-intent nephrectomy [104]. Sunitinib is the only TKI approved in this setting based on the phase 3 trial S-TRAC (NCT00375674) which revealed a statistically significant prolonged disease-free survival over placebo despite the adverse effects of sunitinib [105]. However, this approval was controversial, as the phase 3 trial ASSURE (NCT00326898) showed that sunitinib did not have any survival benefit relative to placebo in the adjuvant setting [106]. Pembrolizumab is the first ICI to provide promising data as an adjuvant therapy, based on the phase 3 trial KEYNOTE-564 (NCT00375674) [107]. In this trial, 994 patients with ccRCC at high risk for recurrence after nephrectomy were randomized to adjuvant pembrolizumab (*n* = 496) or placebo (*n* = 498). Disease-free survival was significant improved in patients who received pembrolizumab (77.3% vs. 68.1%; HR for recurrence or death 0.68; 95% CI, 0.53–0.87; *p* = 0.002). Overall survival data remain immature. Based on these promising results, the FDA granted pembrolizumab in the adjuvant therapy setting priority review in August 2021.

### 4.4. Non-Clear Cell Renal Cell Carcinoma

When ccRCC and nccRCC tumors possess sarcomatoid or rhabdoid features, they are often more aggressive. Clinical implications include shorter time to the development of metastases and decreased overall survival time [108,109]. Because of their rarity and aggressive nature, no definitive treatments exist for these pathologic subtypes [110,111]. 

In single-institution studies, ICIs appear to have better outcomes for sarcomatoid RCC (sRCC) compared to targeted therapies like VEGF inhibitors [112,113]. In a meta-analysis of sRCC in JAVELIN Renal 101, IMmotion151, KEYNOTE-426, and the intermediate/poor patient group of CheckMate 214, the conclusion is support for ICI-driven therapy for these patients over sunitinib [114]. More prospective data are needed for patients with RCC with sarcomatoid and/or rhabdoid features treated with ICIs.

Similarly, for nccRCC, the best treatment options remain unclear due to underrepresentation in clinical trials. Most of the evidence-based data are limited to retrospective studies. Chahoud et al. [115] analyzed 40 patients (including 8 ccRCC patients with rhabdoid features) who received nivolumab at one institution. The mPFS was 4.9 months (95% CI, 3.53–10.27) and mOS was 21.7 months (95% CI, 7.83–NR). In one multi-institutional retrospective study, Koshkin et al. [116] analyzed 41 patients (including 5 patients with sarcomatoid features) with nccRCC who received at least one dose of nivolumab (median treatment duration 3.0 months). The mPFS was 3.5 months (95% CI, 1.9–5.0) and overall survival at 10 months was 68%. In another multi-institutional study, McKay et al. [117] included 43 patients (11 patients with rhabdoid and/or sarcomatoid features) who received PD-1/PD-L1 inhibitors. Overall, eight patients (19%) had an objective response, median time-to-treatment failure was 4.0 months (95% CI, 2.8–5.5), and mOS was 12.9 months (95% CI, 7.4–NR). All these studies underscore the urgent need for prospective studies in this heterogenous population.

KEYNOTE-427 (NCT02853344), as mentioned in a prior section, had a nccRCC cohort that received pembrolizumab monotherapy as first-line treatment. This is one of the first trials with nccRCC patients with published data. For 165 patients with previously untreated advanced nccRCC, the ORR was 26.7%, mPFS was 4.2 months (95% CI, 2.9–5.6), and mOS was 28.9 months (95% CI, 24.3–NR) [97]. These data suggest that immunotherapy may be effective as a first-line treatment for nccRCC.

## 5. Prostate Cancer

Prostate cancer tends to progress more slowly and is usually less lethal than other cancers, but after it metastasizes, the five-year survival rate drops significantly [118]. Metastatic castration-resistant prostate cancer (mCRPC), which is incurable, justifies the need for effective novel therapies. Unlike kidney and bladder cancer, few immunotherapies have been approved for prostate cancer due to disappointing results such as in phase 3 trials where participants received ipilimumab [119,120]. In KEYNOTE-199 (NCT02787005), which included 258 mCRPC patients who previously received docetaxel and endocrine-targeted therapy and were given pembrolizumab, the ORR was only 3% and 5% in two of the study cohorts with response evaluation criteria in solid tumors (RECIST)-measurable disease, but those patients who benefited had a robust response [121]. The limited role for ICIs in prostate cancer could be explained by a lower mutational burden and decreased homing targets for the immune system in the tumor microenvironment [122].

Sipuleucel-T is a vaccine-based therapy that utilizes host antigen-presenting cells (APCs) that are exogenously exposed to prostatic acid phosphatase (PAP) and granulocyte-macrophage colony-stimulating factor (GM-CSF). PAP and GM-CSF are processed by the APCs, and the activated cells are infused into the recipient. T cells recognize the activated APCs and become primed for tumor attack. In the phase 3 IMPACT trial (NCT00065442), 512 mCRPC patients with little to no symptoms were treated with either sipuleucel-T (*n* = 341) or placebo (*n* = 171) [123]. For patients who received sipuleucel-T, there was a longer mOS (25.8 months vs. 21.7 months), and the adjusted hazard ratio for death was 0.78 (95% CI, 0.61–0.98; *p* = 0.03). However, the time to objective disease progression (14.6 weeks vs. 14.4 weeks; HR 0.95; 95% CI, 0.77–1.17; *p* = 0.63) or clinical disease progression (HR 0.92; 95% CI, 0.75–1.12; *p* = 0.40) did not differ significantly between the groups. The data helped lead to FDA approval of sipuleucel-T in patients with asymptomatic or minimally symptomatic mCRPC in April 2010.

Prostate cancer genetics has helped inform treatment. Liquid biopsies from blood samples evaluating circulating tumor DNA have been implemented successfully in a multi-institutional case series of advanced prostate cancer, in which 15 of 460 patients (3.7%) had high levels of microsatellite instability (MSI-H) based on liquid biopsies [124]. In the study, nine patients with mCRPC received pembrolizumab, and three of five patients evaluated had a radiographic response to the treatment.

Pembrolizumab is the one ICI approved for a special group of cancer patients regardless of primary site based on genetic testing. In an initial phase 2 trial (NCT01876511) that enrolled 41 patients with various metastatic cancers (not including any GU cancers) with or without mismatch-repair deficiency, pembrolizumab showed a clinical benefit in patients with mismatch-repair gene deficiency [125]. The authors reported further success from an analysis of 86 patients, which included one prostate cancer patient that had a complete response [126]. Based on promising data from 149 patients in five trials, pembrolizumab was granted accelerated approval in May 2017 to treat MSI-H/dMMR (deficient mismatch repair) disease that had progressed on prior therapy, including unresectable or metastatic prostate cancer [127]. In June 2020, pembrolizumab received accelerated approval for adult and pediatric cancer patients with a high tumor mutational burden (TMB) (≥10 mutations/megabase) that had progressed despite prior treatment, based on results from the phase 2 trial KEYNOTE-158 (NCT02628067) [128]. The trial enrolled 1073 patients with advanced solid tumors which included 102 patients with a high tissue TMB and 688 patients in the non-high tissue TMB group. The ORR was 29% (95% CI, 21–39) in the high tissue TMB cohort compared to 6% (95% CI, 5–8) in the non-high tissue TMB cohort.

The field of immunotherapy in prostate cancer has expanded to include chimeric antigen receptor (CAR-T) cells and bispecific T-cell engagers (BiTe). In pre-clinical studies, engineered CAR-T cells were shown to effectively combat prostate cancer by targeting prostate specific membrane antigen (PSMA) [129]. More recently, there have been pre-clinical studies combining CAR-T cells with chemotherapy (docetaxel) that showed antitumor activity in a xenograft model [130]. In humans, there was a small phase 1 study where anti-PSMA CAR-T cells were infused into five patients with metastatic prostate cancer, and there was a partial reduction in PSA in two patients [131]. Pasotuxizumab, a bispecific T-cell engager developed to target prostate cancer cells via PSMA, was given to 47 patients with advanced prostate cancer either subcutaneously or through continuous IV (cIV) in a phase 1 study (NCT01723475) [132]. In the cIV cohort, there was a dose-dependent reduction in the level of PSA in 14 out of 16 patients, illustrating that BiTe may be a therapy option for CRPC, though further clinical studies are necessary.

For neuroendocrine mCRPC, there is a Category 3 recommendation from the National Comprehensive Cancer Network that the combination of atezolizumab, carboplatin, and etoposide can be used as a treatment strategy. This is an extrapolation from a study that showed that this treatment combination resulted in longer OS and PFS in patients with extensive-stage small-cell lung carcinoma [133]. In the phase 2 trial DART SWOG S1609 (NCT02834013), 32 patients with non-pancreatic neuroendocrine tumors were enrolled, including two patients with prostate cancer, who received ipilimumab and nivolumab [134]. Patients with high-grade tumors had a significantly better overall response rate compared to patients with low/intermediate grade tumors (*p* = 0.004).

## 6. Penile/Testicular/Adrenocortical Cancer

With rare GU tumors including penile, testicular, and adrenocortical cancers, there are indications for immunotherapy in advanced disease that has responded poorly to prior treatment in specific circumstances. Like prostate cancer with MSI-H/dMMR, pembrolizumab can be used for these cancers with MSI-H/dMMR [127]. In a case series from a phase 2 basket trial (NCT02721732), pembrolizumab was effective in achieving partial response in one patient with penile SCC and MSI-H, while two other patients with microsatellite stability progressed [135].

Overall, sufficient data on immunotherapy are lacking for these cancers. Results have been limited to phase 1/2 trials, and so far, ICIs have not shown to be very effective in treating these tumors. In a phase 2 study that enrolled 12 patients with relapsed metastatic germ-cell tumors (11 of testicular origin) refractory to first line and salvage chemotherapy who were treated with pembrolizumab, there was no significant partial response or complete response in any of the patients [136]. In the phase 2 trial APACHE (NCT03081923), 22 patients were randomized to receive either durvalumab with tremelimumab (*n* = 11) versus durvalumab alone (*n* = 11), and nearly all the patients had progressive disease regardless of TMB and PD-L1 expression [137]. More trials have enrolled patients with tumors testing other ICIs, including a phase 1 trial (NCT02496208) evaluating optimal dosing for cabozantinib, nivolumab, and ipilimumab that enrolled six patients with germ cell tumor and one patient with Sertoli cell tumor [138].

Likewise, adrenocortical carcinomas (ACC) are a heterogeneous group of characteristically late-presenting tumors for which treatment options remain limited. There are data from four phase 2 trials in ACC for the efficacy of pembrolizumab (NCT02721732 and NCT02673333) [139,140], nivolumab (NCT02720484) [141], and nivolumab with ipilimumab (NCT03333616) [142]. These phase 2 trials all demonstrated ICIs possess some activity against ACC. The largest ICI trial is the phase 1b trial JAVELIN Solid Tumor (NCT01772004) of avelumab which enrolled 50 patients, of which 25 patients remained on the adrenolytic mitotane [143]. With a median follow-up time of 16.5 months, the disease control rate was 48.0%, OS was 10.6 months (95% CI, 7.4–15.0), and mPFS (95% CI, 1.4–4.0) was 2.6 months. ORR was 16.7% among 12 patients with PD-L1+ tumors and 3.3% among 30 patients with PD-L1- tumors. Larger trials involving ICIs are necessary to further examine their efficacy in ACC.

## 7. Potential Predictive Biomarkers for ICIs

Despite the rapid advancements in immunotherapy, many patients who receive ICIs either continue to have their disease progress despite treatment, or their cancer may initially respond to therapy but then relapse. Because of this, there is an urgent need for predictive biomarkers for ICIs. Three promising biomarkers approved by the FDA for patient selection to respond to ICIs, as mentioned in this article, include PD-L1, MSI-H/dMMR, and TMB [144]. Although data support the notion that the presence of these biomarkers may render cancer more susceptible ICIs, conflicting data exist. There are no accepted guidelines, cutoffs, or standard assays for PD-L1 expression, TMB, or MSI-H/dMMR. which vary among different cancers and were studied differently in clinical trials [144,145,146]. Indeed, for ICI clinical trials involving mUC and RCC, cutoffs to determine PD-L1 status for immune cells and/or tumor cells, and the assays used to detect PD-L1 status, varied widely. Presently, these biomarkers are not reliable predictors for ICI efficacy in GU oncology. Several emerging biomarkers currently being studied include mutations in phosphatase, polymerase, and transcription factor proteins, as well as combining multiple biomarkers for improving predictive performance [144,145].

## 8. Future Immune Checkpoint Inhibition Trials

ICIs have made major strides in GU oncology in the past decade. New trials are active and enrolling, aiming to steadily increase the median overall survival and progression-free survival in patients with metastatic GU cancers (Table 4). A large proportion of upcoming immune checkpoint inhibition trials will evaluate for synergistic benefit among ICIs and other treatment strategies. These include EZH2 and arginase inhibitors, hypomethylating agents, novel decoy receptors, and new recombinant human interleukins. Phase 2/3 trials are underway to evaluate ICIs as front-line therapy in combination with tyrosine kinase inhibitors in the GU tumors, building upon the success of this combination in RCC.

## 9. Conclusions

In summary, the field of immunotherapy has rapidly expanded in the past decade, especially involving immune checkpoint inhibition. This has made for groundbreaking strides in the world of GU oncology. ICIs are now at the forefront of cancer therapy, especially in front-line metastatic RCC and in multiple settings of bladder/urothelial cancer. The use of ICIs in prostate cancer, testicular cancer, penile cancer, and adrenocortical cancer has not been as heavily explored, but in certain situations may provide clinical benefit. However, there are still a few setbacks with ICIs, as not all phase 3 trials have been shown to be superior to traditional therapy, and not all long-term responses have remained durable. This suggests that further research is still necessary to determine which patients are most likely to benefit from immunotherapy. Looking towards the future, several more clinical trials are underway in GU oncology evaluating the combination of ICIs with other targeted therapies. The goal is to enhance “precision therapy” to increase overall survival and progression-free survival. With the successes that have stemmed from the intersection of GU oncology and immunotherapy over the last decade, the future of these fields is bright.

## Figures and Tables

**Table 1:** Trials containing immune checkpoint inhibitors that led to Food and Drug Administration (FDA) approval to treat genitourinary cancers.

Trial	Agent(s)	Cancer Subtype and Disease Setting	Description	Original Food and Drug Administration Approval Date	Modifications
NCT02625961 (KEYNOTE-057)	Pembrolizumab monotherapy	Non-muscle-invasive bladder cancer	BCG refractory	January 2020	
NCT02632409 (CheckMate 274)	Nivolumab monotherapy	Muscle-invasive bladder cancer	Adjuvant therapy after radical resection	August 2021	
NCT02951767 (IMvigor210)	Atezolizumab monotherapy	Locally advanced or metastatic cisplatin-ineligible urothelial carcinoma	First-line metastatic	April 2017 (accelerated approval)	June 2018 (stricter guidelines including PD-L1 expression)
NCT02335424 (KEYNOTE-052)	Pembrolizumab monotherapy	Locally advanced or metastatic cisplatin-ineligible urothelial carcinoma	First-line metastatic	August 2021	
NCT03288545 (EV-103/KEYNOTE-869)	Pembrolizumab and enfortumab vedotin	Locally advanced or metastatic cisplatin-ineligible urothelial carcinoma	First-line metastatic	February 2020 (breakthrough designation)	
NCT02603432 (JAVELIN Bladder 100)	Avelumab monotherapy	Locally advanced or metastatic urothelial carcinoma	First-line maintenance after platinum-based chemotherapy	June 2020	
NCT02108652 (IMvigor210)	Atezolizumab monotherapy	Locally advanced or metastatic urothelial carcinoma after platinum therapy	Second-line metastatic	May 2016 (accelerated approval)	Withdrawal in March 2021
NCT02387996 (CheckMate 275)	Nivolumab monotherapy	Locally advanced or metastatic urothelial carcinoma after platinum therapy	Second-line metastatic	February 2017 (accelerated approval)	
NCT02256436 (KEYNOTE-045)	Pembrolizumab monotherapy	Locally advanced or metastatic urothelial carcinoma after platinum therapy	Second-line metastatic	May 2017	
NCT01772004 (JAVELIN Solid Tumor)	Avelumab monotherapy	Locally advanced or metastatic urothelial carcinoma after platinum therapy	Second-line metastatic	May 2017 (accelerated approval)	
NCT01693562 (Study 1108)	Durvalumab monotherapy	Locally advanced or metastatic urothelial carcinoma after platinum therapy	Second-line metastatic	May 2017 (accelerated approval)	Withdrawal in February 2021
NCT02853331 (KEYNOTE-426)	Pembrolizumab and axitinib	Metastatic renal cell carcinoma	First-line metastatic	April 2019	
NCT02684006 (JAVELIN Renal 101)	Avelumab and axitinib	Metastatic renal cell carcinoma	First-line metastatic	May 2019	
NCT0281186 (CLEAR)	Lenvatinib and pembrolizumab	Metastatic renal cell carcinoma	First-line metastatic	August 2021	
NCT03141177 (CheckMate 9ER)	Nivolumab and cabozantinib	Metastatic renal cell carcinoma	First-line metastatic	January 2021	
NCT02231749 (CheckMate 214)	Nivolumab and ipilimumab	Metastatic renal cell carcinoma	First-line metastatic	April 2018	
NCT01668784 (CheckMate 025)	Nivolumab	Metastatic renal cell carcinoma previously treated with angiogenic inhibitor	Second-line metastatic	November 2015	
NCT01876511	Pembrolizumab	Tumors with high microsatellite instability or deficiency in mismatch repair refractory to other treatments	Progression on at least one prior systemic therapy	May 2017 (accelerated approval)	
NCT02628067 (KEYNOTE-158)	Pembrolizumab	Tumors with high mutational burden refractory to other treatments	Progression on at least one prior systemic therapy	June 2020 (accelerated approval)	

**Table 2:** Trials with immune checkpoint inhibitors at various stages of bladder cancer leading to approval for use.

Trial	Phase	Agent(s)	Bladder Cancer Stage	mOS	mPFS	ORR
NCT02625961 (KEYNOTE-057) [11]	1	Pembrolizumab monotherapy	Non-muscle-invasive bladder cancer after BCG treatment	NR	NR	- *
NCT02632409 (CheckMate 274) [12]	3	Adjuvant nivolumab vs. control	Muscle-invasive bladder cancer after radical surgery	-	-	- **
NCT02603432 (JAVELIN Bladder 100) [13]	3	Avelumab monotherapy vs. control	Locally advanced or metastatic urothelial carcinoma	21.4 vs. 14.3 months (HR 0.69; 95% CI, 0.56–0.86; *p* = 0.001)	3.7 vs. 2.0 months (HR 0.62; 95% CI, 0.52–0.75; *p* < 0.001)	9.7 vs. 1.4% (NA)
NCT02951767 (IMvigor210) [14,15]	2	Atezolizumab monotherapy	Locally advanced or metastatic cisplatin-ineligible urothelial cancer	16.3 months (95% CI, 10.4–24.5)	2.7 months (95% CI, 2.1–4.2)	24% (95% CI, 16–32)
NCT02335424 (KEYNOTE-052) [16,17]	2	Pembrolizumab monotherapy	Locally advanced or metastatic cisplatin-ineligible urothelial cancer	11.3 months (95% CI, 9.7–13.1)	2.2 months (95% CI, 2.1–3.4)	28.6% (95% CI, 24.1–33.5)
NCT03288545 (EV-103/KEYNOTE-869) [18]	1/2	Pembrolizumab and enfortumab vedotin	Locally advanced or metastatic cisplatin-ineligible urothelial cancer	-	12.3 months (95% CI, 7.98–NR)	73.3% (95% CI, 58.1–85.4)
NCT02108652 (IMvigor210) [19]	2	Atezolizumab monotherapy	Locally advanced or metastatic urothelial carcinoma after platinum therapy	7.9 months (95% CI, 6.6–9.3)	2.1 months (95% CI, 2.1–2.1)	15% (95% CI, 11–19)
NCT02387996 (CheckMate 275) [20,21]	2	Nivolumab monotherapy	Locally advanced or metastatic urothelial carcinoma after platinum therapy	8.6 months (95% CI, 6.1–11.3)	1.9 months (95% CI, 1.9–2.3)	20.7% (95% CI, 16.1–26.1)
NCT02256436 (KEYNOTE-045) [22,23]	3	Pembrolizumab monotherapy vs. control	Locally advanced or metastatic urothelial carcinoma after platinum therapy	10.1 vs. 7.3 months (HR 0.70; 95% CI, 0.57–0.85; *p* < 0.001)	2.1 vs. 3.3 months (HR 0.96; 95% CI, 0.79–1.16; *p* = 0.313)	21.1 vs. 11.0% (NA)
NCT01772004 (JAVELIN Solid Tumor) [24,25]	1	Avelumab monotherapy	Locally advanced or metastatic urothelial carcinoma after platinum therapy	7.0 months (95% CI, 5.9–8.5)	1.6 months (95% CI, 1.4–2.7)	16.5% (95% CI, 12.1–21.8)
NCT01693562 (Study 1108) [26,27]	1	Durvalumab monotherapy	Locally advanced or metastatic urothelial carcinoma after platinum therapy	18.2 months (95% CI, 8.1–NE)	1.5 months (95% CI, 1.4–1.9)	17.8% (95% CI, 12.7–24.0)

Abbreviations: mOS, median overall survival; mPFS, median progression-free survival; ORR, objective response rate; BCG, Bacillus Calmette–Guérin. * Complete response rate was the primary endpoint: 40.6%. ** Median disease-free survival was the primary endpoint: 20.8 months vs. 10.8 months in the intention-to-treat group.

**Table 3:** Trials with immune checkpoint inhibitors for metastatic renal cell carcinoma (RCC) leading to approval for use.

Trial	Phase	Agent(s)	RCC Stage	mOS	mPFS	ORR (*p*-Value)
NCT02231749 (CheckMate 214) [76,77]	3	Nivolumab and ipilimumab versus sunitinib	Metastatic renal cell carcinoma	48.1 vs. 26.6 months (HR 0.65; 95% CI, 0.54–0.78) *	11.2 vs. 8.3 months (HR 0.74; 95% CI, 0.62–0.88) *	41.9 vs. 26.8% (*p* < 0.0001) *
NCT02853331 (KEYNOTE-426) [78,79]	3	Pembrolizumab with axitinib versus sunitinib	Metastatic renal cell carcinoma	NR vs. 35.7 months (HR 0.68; 95% CI, 0.55–0.85; *p* = 0.0003)	15.4 vs. 11.1 months (HR 0.71; 95% CI, 0.60–0.84; *p* < 0.0001)	60 vs. 40% (*p* < 0.001)
NCT02501096 (CLEAR) [80]	3	Lenvatinib with pembrolizumab versus sunitinib	Metastatic renal cell carcinoma	NR vs. NR (HR 0.66; 95% CI, 0.49–0.88; *p* = 0.005)	23.9 vs. 9.2 months (HR 0.39; 95% CI, 0.32–0.49; *p* < 0.001)	71.0% vs. 36.1% (NA)
NCT03141177 (CheckMate 9ER) [81]	3	Nivolumab and cabozantinib versus sunitinib	Metastatic renal cell carcinoma	NR vs. NR (HR 0.60; 98.89% CI, 0.40–0.89; *p* = 0.001)	16.6 vs. 8.3 months (HR 0.51; 95% CI, 0.41–0.64; *p* < 0.001)	55.7% vs. 27.1% (*p* < 0.001)
NCT02684006 (JAVELIN Renal 101) [82,83]	3	Avelumab with axitinib versus sunitinib	Metastatic renal cell carcinoma	NR vs. NR (HR 0.80; 95% CI, 0.62–1.03; *p* = 0.0392)	13.3 vs. 8.0 months (HR 0.69; 95% CI, 0.57–0.83; *p* < 0.0001)	52.5% vs. 27.3% (NA)
NCT02420821 (IMmotion151) [84]	3	Atezolizumab with bevacizumab versus sunitinib	Metastatic renal cell carcinoma	33.6 vs. 34.9 months (HR 0.93; 95% CI, 0.76–1.14; *p* = 0.4751)	11.2 vs. 8.4 months (HR 0.83; 95% CI, 0.70–0.97; *p* = 0.0219)	37% vs. 33% (NA)
NCT01668784 (CheckMate 025) [85,86]	3	Nivolumab versus everolimus	Metastatic renal cell carcinoma previously treated with angiogenic inhibitor	25.8 vs. 19.7 months (HR 0.73; 95% CI, 0.62–0.85; *p* < 0.001)	4.2 vs. 4.5 months (HR 0.84; 95% CI, 0.72–0.99; *p* = 0.0331) **	23% vs. 4% (*p* < 0.001)

Abbreviations: mOS, median overall survival; mPFS, median progression-free survival; ORR, objective response rate; NA, not available. * Poor/intermediate risk group only. ** On extended follow-up, the PFS favored nivolumab over everolimus.

**Table 4:** Ongoing immune checkpoint inhibitor trials for metastatic genitourinary cancers.

Trial	Phase	Setting	Intervention	Comparator	Sample Size
Bladder					
NCT04223856	3	1st line	Pembrolizumab, EV	Gemcitabine, cisplatin	760
NCT03682068	3	1st line	Durvalumab, tremelimumab, chemotherapy	Chemotherapy	1292
NCT03036098	3	1st line	Nivolumab, ipilimumab or nivolumab, chemotherapy	Chemotherapy	1290
NCT03898180	3	1st line	Pembrolizumab, lenvatinib	Pembrolizumab, placebo	694
NCT03601455	2	1st line	Durvalumab, tremelimumab, radiation	Durvalumab, radiation	13
NCT03606174	2	1st line	Nivolumab, pembrolizumab, EV, sitravatinib		425
NCT03534804	2	1st line	Pembrolizumab, cabozantinib		39
NCT03785925	2	1st line	Nivolumab, bempegaldesleukin		192
NCT03288545	1/2	1st or 2nd line	Pembrolizumab, EV, chemotherapy		457
NCT03854474	1/2	1st or 2nd line	Pembrolizumab, tazemetostat		30
NCT04045613	1/2	1st or ≥2nd line	Atezolizumab, derazantinib		272
NCT04953104	2	≥2nd line	Nivolumab		30
NCT04101812	2	2nd line	PD-1 ICI, doxorubicin	PD-1 ICI	60
NCT02717156	2	2nd line	Pembrolizumab, sEphB4-HSA		60
NCT04004442	1/2	2nd line	Avelumab, AVB-S6-500		31
NCT03179943	2	≥2nd line	Atezolizumab, guadecitabine		21
NCT03547973	2	≥2nd line	Pembrolizumab, SG, avelumab, cisplatin		321
NCT03513952	2	≥2nd line	Atezolizumab, CYT107		54
Kidney					
NCT03793166	3	1st line	Nivolumab, ipilimumab then nivolumab, cabozantinib	Nivolumab, ipilimumab then nivolumab	1046
NCT03937219	3	1st line	Nivolumab, ipilimumab, cabozantinib	Nivolumab, ipilimumab, placebo	840
NCT03729245	3	1st line	Nivolumab, bempegaldesleukin	Sunitinib or cabozantinib	623
NCT03977571	3	1st line **	Nivolumab, ipilimumab, nephrectomy	Nivolumab, ipilimumab	400
NCT04510597	3	1st line **	ICI with/without axitinib, nephrectomy	ICI	364
NCT04338269	3	2nd line **	Atezolizumab, cabozantinib	Cabozantinib	500
NCT04987203	3	2nd or 3rd line	Nivolumab, tivozanib	Tivozanib	326
NCT03117309	2	1st line **	Nivolumab and salvage nivolumab, ipilimumab		134
NCT04704219	2	1st line *	Pembrolizumab, lenvatinib		152
NCT04267120	2	1st line *	Pembrolizumab, lenvatinib		34
NCT03075423	2	1st line *	Nivolumab, ipilimumab	Sunitinib	306
NCT04644432	2	1st line *	Pembrolizumab or nivolumab		30
NCT03177239	2	≥1st line *	Nivolumab, then nivolumab, ipilimumab		85
NCT04413123	2	≥1st line *	Nivolumab, ipilimumab, cabozantinib		40
NCT03274258	2	≥1st line *	Nivolumab, ipilimumab		10
NCT03595124	2	TFE/tRCC	Nivolumab, axitinib	Nivolumab	70
NCT04385654	2	Neoadjuvant *	Toripalimab, axitinib		40
NCT02724878	2	≥1st line *	Atezolizumab, bevacizumab		65
NCT03297593	2	1st or 2nd line	Nivolumab, ipilimumab		74
NCT04698213	2	1st line	Avelumab, axitinib		75
NCT04338269	3	≥2nd line **	Atezolizumab, cabozantinib	Cabozantinib	500
NCT03469713	2	2nd or 3rd line	Nivolumab, SBRT		69
NCT03149822	1/2	ICI eligible	Pembrolizumab, cabozantinib		45
NCT04758507	1/2	ICI eligible	ICI, microbiota transplant	ICI, placebo transplant	50
Prostate					
NCT04191096	3	mHSPC	Pembrolizumab, enzalutamide, ADT	Placebo, enzalutamide, ADT	1232
NCT03879122	2/3	mHSPC	Nivolumab, ipilimumab, docetaxel, ADT	Docetaxel, ADT	135
NCT04126070	2	mHSPC	Nivolumab, docetaxel, ADT		60
NCT04633252	1/2	mCRPC, mHSPC	M7824, M9241, docetaxel, ADT		86
NCT04104893	2	mCRPC	Pembrolizumab		30
NCT03506997	2	mCRPC	Pembrolizumab		100
NCT04116775	2	mCRPC	Pembrolizumab, microbiota transplant, enzalutamide		32
NCT03406858	2	mCRPC	Pembrolizumab, HER2Bi-armed activated T cells		33
NCT03040791	2	mCRPC	Nivolumab		38
NCT04717154	2	mCRPC	Nivolumab, ipilimumab		75
NCT03570619	2	mCRPC	Nivolumab, ipilimumab		40
NCT03554317	2	mCRPC	Nivolumab, BAT		44
NCT04089553	2	mCRPC	Durvalumab, oleclumab, AZD4635		58
NCT04926181	2	mCRPC	Cetrelimab, apalutamide		24
NCT03910660	1/2	mCRPC	Pembrolizumab, talabostat mesylate		40
NCT02861573	1/2	mCRPC	Pembrolizumab combination therapies		1000
NCT02484404	1/2	mCRPC	Olaparib, cediranib, durvalumab		384
NCT04381832	1/2	mCRPC	Zimberelimab combination therapies		140
NCT03673787	1/2	mCRPC	Atezolizumab, ipatasertib		87
NCT05000294	1/2	mCRPC	Atezolizumab, tivozanib		29
Rare tumors					
NCT03427411	2	HPV-associated disease	M7824		57
NCT02834013	2	2nd line	Ipilimumab, nivolumab		818
NCT02721732	2	≥2nd line	Pembrolizumab		225
NCT03333616	2	Advanced disease	Ipilimumab, nivolumab		100
NCT03866382	2	Advanced disease	Cabozantinib, ipilimumab, nivolumab		224
NCT04400474	2	Advanced disease	Cabozantinib, atezolizumab		144
NCT04187404	1/2	Advanced disease	Nivolumab, EO2401		60

Abbreviations: EV, enfortumab vedotin; SG, sacituzumab govitecan; ICI, immune checkpoint inhibitor; SBRT, stereotactic body radiation therapy; ADT, androgen deprivation therapy; BAT, bipolar androgen therapy; TFE/tRCC, transcription factor E3/translocation morphology renal cell carcinoma; mHSPC, metastatic hormone-sensitive prostate cancer; mCRPC: metastatic castration-resistant prostate cancer; *, non-clear cell renal cell carcinoma patients enrolled only; **, both clear cell and non-clear cell renal cell carcinoma patients enrolled.
